# Analyzing the relative importance of habitat quantity and quality for boosting pollinator populations in agricultural landscapes

**DOI:** 10.1111/cobi.14317

**Published:** 2024-06-25

**Authors:** Thijs P. M. Fijen, Gabriella A. Bishop, Cristina Ganuza, Jeroen Scheper, David Kleijn

**Affiliations:** ^1^ Plant Ecology and Nature Conservation Group Wageningen University & Research Wageningen The Netherlands; ^2^ Department of Animal Ecology and Tropical Biology Julius‐Maximilians‐University Würzburg Würzburg Germany

**Keywords:** agricultural landscapes, conservation, habitat quality, habitat quantity, hoverflies, pollinator populations, seminatural habitat, wild bees, abejas silvestres, calidad del hábitat, cantidad de hábitat, conservación, hábitat seminatural, paisaje agrícola, poblaciones de polinizadores, sírfidos

## Abstract

To increase pollinator populations, international policy targets minimum levels of seminatural habitat cover, but it is unknown whether improving the quality of existing habitats could bring similar benefits without the need of reducing cropland area. Using data we collected in 26 Italian agricultural landscapes during the entire flying season, we explored the relative importance of habitat quantity (seminatural habitat cover) and quality (flower availability) on pollinator densities in seminatural habitats. We obtained transect‐based counts and estimated the effect of habitat quantity (proportion of seminatural habitat) and quality (flower cover and richness) on wild bee and hoverfly densities. We used the relationships revealed in the data to simulate pollinator population sizes in landscapes with varying habitat quantity and quality. Wild bee densities were only related to flower availability, whereas hoverfly densities were additionally related to seminatural habitat cover. We found that in complex agricultural landscapes (above 15% seminatural habitat cover), improving habitat quality increased pollinator populations more effectively than increasing habitat quantity. However, increasing habitat quantity was by far the most effective approach for boosting pollinator populations in simple landscapes.

## Abstract (es)

Análisis de la importancia relativa de la cantidad y calidad del hábitat para incrementar las poblaciones de polinizadores en los paisajes agrícolas

**Resumen:**

Las políticas internacionales buscan que existan niveles mínimos de cobertura seminatural del hábitat para incrementar las poblaciones de polinizadores y se desconoce si mejorar la calidad de los hábitats existentes podría brindar beneficios similares sin tener que reducir el área de cultivo. Usamos datos recolectados en 26 paisajes agrícolas de Italia durante la temporada de vuelo para analizar la importancia relativa de la cantidad (cobertura de hábitat seminatural) y calidad (disponibilidad de flores) del hábitat para la densidad de polinizadores en los hábitats seminaturales. Obtuvimos conteos basados en transectos y estimamos el efecto de la cantidad (proporción del hábitat seminatural) y calidad (riqueza y cobertura de flores) del hábitat sobre la densidad de las abejas silvestres y los sírfidos. Usamos la relación revelada por los datos para simular el tamaño poblacional de los polinizadores en los paisajes con diferente calidad y cantidad de hábitat. La densidad de las abejas silvestres sólo se relacionó con la disponibilidad de flores cuando la densidad de sírfidos se relacionó con la cobertura del hábitat seminatural. Descubrimos que en los paisajes agrícolas complejos (por encima del 15% de cobertura de hábitat seminatural) cuya calidad mejoraba, las poblaciones de polinizadores incrementaban de manera más eficiente que cuando se mejoraba la cantidad. Sin embargo, incrementar la cantidad del hábitat fue por mucho la estrategia más efectiva para acrecentar las poblaciones de polinizadores en paisajes simples.

## INTRODUCTION

Global reports of declining insect populations have raised concerns about food security because the majority of crops are at least partially dependent on insects for pollination (Klein et al., 2007). This has inspired a plethora of international (e.g., U.S. Conservation Reservation program, European Union Agri‐Environmental Schemes), national (e.g., All‐Ireland Pollinator Plan, Dutch National Pollinator Strategy), and local initiatives to set up monitoring schemes and to protect pollinators and the ecosystem services they deliver. Because landscape simplification (i.e., removal of seminatural habitat elements) is a major driver of pollinator declines in agricultural landscapes (Dainese et al., 2019), several policy targets aim to achieve a minimum amount of seminatural habitat cover in agricultural landscapes. For example, the EU Biodiversity Strategy for 2030 states that at least 10% of agricultural area should be occupied with high‐diversity landscape features (European Commission & Directorate‐General for Environment, 2021), and scientists claim that 20% seminatural habitat cover is needed to achieve effective, sustainable working landscapes (Garibaldi et al., 2020; Kremen & Merenlender, 2018).

The call for these thresholds for seminatural habitat cover is based on a large evidence base showing that increasing the cover of seminatural habitat generally leads to enhanced crop pollination services (Dainese et al., 2019). Insects that pollinate crops depend on seminatural habitats for floral resources when crops are not flowering, and arable fields are generally unsuitable for nesting and shelter (Fijen et al., 2019; Martínez‐Núñez et al., 2022; Schellhorn et al., 2015). When crops start flowering, they attract pollinators from nearby seminatural habitats, particularly those that preferentially forage on crops (Fijen et al., 2019; Kleijn et al., 2015). With more seminatural habitat present in the landscape, the population size of potential crop pollinators will be larger, resulting, all else being equal, in higher visitation rates and often better pollination (Dainese et al., 2019). Interestingly, although pollinator communities in crops are almost invariably influenced by the amount of seminatural habitat in the surroundings, in the seminatural habitats themselves they seem to be little affected by seminatural habitat cover in the landscape (Meyer et al., 2009), mass‐flowering crops (Martínez‐Núñez et al., 2022), or the land‐use intensity of adjacent grasslands (Li et al., 2020). This implies that the number of pollinators that can persist in an agricultural landscape is a straight‐forward function of the amount of seminatural habitat (Redhead et al., 2018).

However, in intensive agricultural areas, increasing the seminatural habitat cover for pollinator conservation and crop pollination can only be done by converting productive land to nonproductive land. This leads to high opportunity costs and therefore sees little uptake (Kleijn et al., 2019). There is an increasing interest in understanding how pollinator‐enhancing measures related to the quality and not quantity of seminatural habitat could increase the provision of ecosystem services on farmland (Albrecht et al., 2020; Mei et al., 2021). The quality of seminatural habitat in agricultural landscapes is generally poor (Cole et al., 2020), so increasing the quality of these existing habitats could be an effective alternative to increasing habitat quantity, which often has lower associated costs (Mody et al., 2020; Phillips et al., 2020) and therefore higher acceptance by farmers. However, whether and when it would be more effective to improve quality rather than quantity of seminatural habitat has never been tested (Jauker et al., 2009; Meyer et al., 2009).

Ecological theory predicts that the quality of pollinator habitat depends on the amount of required resources for pollinators that the habitat provides. The needs of pollinators, such as bees and hoverflies, include sites for reproduction (i.e., nesting sites), food for self‐maintenance (mainly nectar), and food for their larvae (e.g., pollen, prey, decaying organic matter) (Rotheray & Gilbert, 2011; Westrich, 1996). Because most pollinators nest in the soil or in undisturbed vegetation (Cane & Neff, 2011; Harmon‐Threatt, 2020; Howlett et al., 2021), nesting site availability is probably best captured by the quantity of nonproductive habitat (Rotheray & Gilbert, 2011; Roulston & Goodell, 2011), although understanding of the role of nesting site availability in determining population size is limited (Albrecht et al., 2023; Tschanz et al., 2022). Additionally, different species use markedly different substrates for reproduction, such as soil, rodent nests, or hollow plant parts, making it challenging to come up with simple, effective indicators of the reproductive quality of habitats for pollinators as a group. However, all pollinator species require flowers to complete their life cycles, and flower availability (i.e., abundance and richness) is generally positively related to pollinator abundance and richness (Albrecht et al., 2020; Bishop et al., 2024; Carré et al., 2009; Segre et al., 2023). Flower availability is therefore most likely a good indicator of habitat quality for insect pollinators.

Ultimately, the size of pollinator populations at the landscape scale is then determined by the combined effects of habitat quantity and quality. Population sizes can therefore be estimated by multiplying the density of pollinators in pollinator habitat by the total amount of pollinator habitat (Kleijn et al., 2018). This means that if habitat quality is high, the same population size can be achieved with much less habitat than when habitat quality is low. Whether this hypothesis holds in real‐world landscapes and how much more habitat with low‐quality habitat is needed to achieve the same population sizes as with habitats of high‐quality depend on the relative importance of habitat quantity and quality for pollinator densities and the landscape context (Kleijn et al., 2018).

These relationships may furthermore differ among different groups of pollinators because of differences in life‐history strategies. Bees and hoverflies are the most important groups of insect crop pollinators (Rader et al., 2016). Bees are central‐place foragers (Westrich, 1996), which makes them reliant on the amount and quality of food that is within flight range of their nest (Larsson & Franzén, 2007; Westrich, 1996). Hoverflies are not bound to a nesting site and can therefore move more easily from low‐ to high‐quality areas (Jauker et al., 2009; Kleijn & van Langevelde, 2006; Meyer et al., 2009) or to flowering crops (Fijen et al., 2019). Furthermore, different hoverfly species have distinctly varied oviposition requirements; for example, some species oviposit in decaying organic matter and others in aphid colonies (Howlett et al., 2021). Such differences in life history between bees and hoverflies may also affect how their populations respond to changes in habitat quantity and quality.

We assessed the relative importance of habitat quality and quantity for pollinator population size (bees and hoverflies, separately) using a data set comprising of 587 pollinator transect counts collected in 26 agricultural landscapes in southern Italy during the entire flying season (8 sampling rounds). We first examined the relative importance of habitat quantity (i.e., seminatural habitat cover) and quality (i.e., flower availability) for pollinator densities in seminatural habitats and whether the relative importance differed for wild bees and hoverflies. Second, we examined how pollinator populations at the landscape scale respond to improving habitat quantity and quality. Third, we determined the landscape context in which it was more effective to improve habitat quality than to improve habitat quantity.

## METHODS

### Study area

This study was carried out in an area of approximately 1400 km^2^ in southern Italy that consisted of agricultural landscapes dominated by wheat cultivation and a wide variety of other (mass‐flowering) crops. In 2018, we selected 26 agricultural landscapes of 750‐m radius based on initial assessments of seminatural habitat cover on Google Earth aerial images. Final landscapes represented a gradient in seminatural habitat cover (0.35–70%, mean [SD] = 30.0 [22.0]) and in number of mass‐flowering crop types (0–8) (e.g., leek [*Allium porrum*], onion [*Allium cepa*], faba bean [*Vicia faba*]) (details in Martínez‐Núñez et al. [2022]). Landscapes were well separated from each other (mean [SD] = 19 km [18]), except for one landscape pair for which the borders of the landscape slightly overlapped. However, because their radii were above the mean maximum foraging range of most wild bees, ∼200–300 m (Zurbuchen et al., 2010), we decided to keep that landscape pair in the analyses.

### Pollinator surveys

We surveyed all bees and hoverflies in each landscape every 2 weeks from the end of March up to the end of July 2018, amounting to a total of 8 rounds of sampling. For this, we used standardized transects of 150 m^2^ (generally 150‐m long and 1‐m wide), subdivided in 3 consecutive subtransects of 50 m^2^ to spread sampling effort evenly over the transect. In each transect, we counted pollinators for 15 min of pure searching time (i.e., 5 min per subtransect). Species not identified to species level on the wing were caught, killed, and stored for later identification to species or morphospecies level. A specific permit to do so was not required. Species richness was not a focus of this study. Honeybees (*Apis mellifera*) were counted but excluded from the analyses because their abundance depended largely on the presence of honeybee hives. All transects were located in a focal area of about 200 × 25 m. On each visit, we selected the most flower‐rich patch within that focal area to follow the pollinators as they tracked changes in local floral resources.

Because different types of pollinator habitats can attract different parts of the pollinator community, we surveyed both herbaceous and woody seminatural habitats, which are the 2 most common and contrasting pollinator habitats in this area (Martínez‐Núñez et al., 2022). Each landscape had 2 transects in herbaceous habitats (separated by at least 200 m), mostly consisting of flower‐rich roadside verges, crop field edges, or extensive grasslands. Woody seminatural habitat, such as hedgerows and forest edges, were surveyed depending on availability to increase the accuracy of pollinator density estimates in pollinator habitat. Seventeen of the 26 landscapes had flowering woody vegetation, and in those landscapes, we surveyed transects proportionally to the amount of flowering woody seminatural habitat (1–2 per landscape per round). In each round, we also noted which insect‐pollinated crop was flowering in each landscape to check whether this influenced pollinator densities in the seminatural habitats (Fijen et al., 2019).

Surveys were conducted when temperatures were above 18°C on sunny and calm days (<5 bft wind) roughly between 08:00 and 17:00 (Fijen & Kleijn, 2017). We took care to balance and alternate morning and afternoon visits to landscapes and varied the order of landscape visits, while keeping a minimum of at least 1 week between visits (range 8–20 days, mean [SD] = 13.2 days [1.8] between visits).

### Seminatural habitat quantity and quality

We used the total seminatural habitat cover as a proxy for habitat quantity in the landscapes. We first estimated seminatural habitat cover (e.g., grasslands, road side verges, hedgerows, and forests) with Google Earth aerial images, followed by ground truthing. We considered all areas that were not built on and that were uncultivated for over 1 year seminatural habitats.

We used flower cover and flower richness as proxies for habitat quality. Flower cover of each flowering plant species was visually estimated immediately after each pollinator survey with increasing precision as flower cover decreased (i.e., 10% cover with 1% precision, and 1% cover with 0.1% precision). Estimations of flower cover of <0.05% (250 cm^2^ per subtransect) were set at a fixed level of 0.025%. Total flower cover was then calculated by summing the flower cover of all species over the entire transect, and total flower diversity was calculated as the total number of unique flowering species per transect.

### Analyses

To examine the relationships between wild bee and hoverfly densities and our proxies for seminatural habitat quantity and quality, we first calculated average densities (wild bees and hoverflies separately), average flower cover, and average flower richness per transect (together comprising habitat quality), per round, and per landscape. We then used linear mixed‐effects models (function glmmTMB in R‐package glmmTMB) (Brooks et al., 2017) to test whether wild bee and hoverfly densities were related to the cover of seminatural habitat in the landscape (percentage), average flower cover in the transect (percentage), average flower richness in the transect (number of flowering species), and presence of a flowering mass‐flowering crop in the landscape (yes or no). We included the 2‐way interaction between seminatural habitat cover and presence of a flowering insect‐pollinated crop because the attraction to crops may depend on seminatural habitat cover (Holzschuh et al., 2016; Riggi et al., 2023). To account for the variation associated between sampling rounds (Martínez‐Núñez et al., 2022) and within landscapes, we included round and landscape identity as separate random factors. Normality of residuals were improved by log_10_ transformations of average wild bee densities and average flower cover and log_10_(+1) transformations of hoverfly densities. We assessed significance of effects with likelihood‐ratio tests. We removed nonsignificant interactions from the models to be able to interpret main effects (Grueber et al., 2011) but retained all main effects because we had clear hypotheses as to why they should be included in the model (Harrell, 2015) and to be able to extrapolate to the landscape scale (see below). Variance inflation factors of all models were checked and were all below 1.5. For visualization purposes, response variables were back transformed after analyses.

After establishing the final models for wild bees and hoverflies, we used these models to predict pollinator densities in different combinations of habitat quantity and quality within the observed ranges. We divided the range of observed seminatural habitat cover in 15 steps of 5% (0.35%, 5%, 10%, … 70%) and both flower cover and richness variables in 20 steps of 5% along the entire observed range (hereafter flower availability). We then created a matrix of model‐predicted densities for each of the combinations among the 15 levels of seminatural habitat cover and the 20 levels of flower availability while keeping potential effects of mass‐flowering crop on pollinator densities constant (crop flowering = no; including a predicted mean interaction or setting crop flowering to yes slightly increased the relative importance of habitat quantity) and not including random effects. In the next step, we extrapolated these predicted densities at each combination of habitat quality and quantity to the landscape level by first back transforming the estimates, dividing the predicted densities by the transect cover to get densities per square meter, and finally multiplying these densities by the proportion of seminatural habitat cover in the entire landscape (seminatural habitat proportion × ϖ × 750^2^). This resulted in estimates of wild bee and hoverfly population sizes at the landscape scale for each combination of habitat quantity and quality. Finally, based on these results, we calculated the threshold at which landscape context would be more beneficial to improve habitat quality or habitat quantity by calculating population increase with one‐step increase in habitat quality (+5%) divided by the population increase with one‐step increase in habitat quantity (+5%), which resulted in a quality:quantity ratio. This threshold analyses implies that improving habitat quality and quantity are equally feasible, which is highly questionable given the costs of taking land out of production. We therefore also calculated thresholds for 2:1 (+10%:+5% quality:quantity) and 3:1 (+15%:+5%) step ratios. These ratios thus illustrated a situation in which one‐step increase in habitat quantity (+5%) is as feasible as increases of 2 or 3 steps in habitat quality (+10% and +15%, respectively).

## RESULTS

We counted 8444 wild bees and 5151 hoverflies in a total of 408 herbaceous seminatural habitat transects and 179 woody seminatural habitat transects (mean [SD] = 2.8 transects per round per landscape [0.8]). Transects had on average 4.6% flower cover [8.1] (geometric mean 2.0%) and 12.8 flowering plant species [5.8].

**FIGURE 1:**
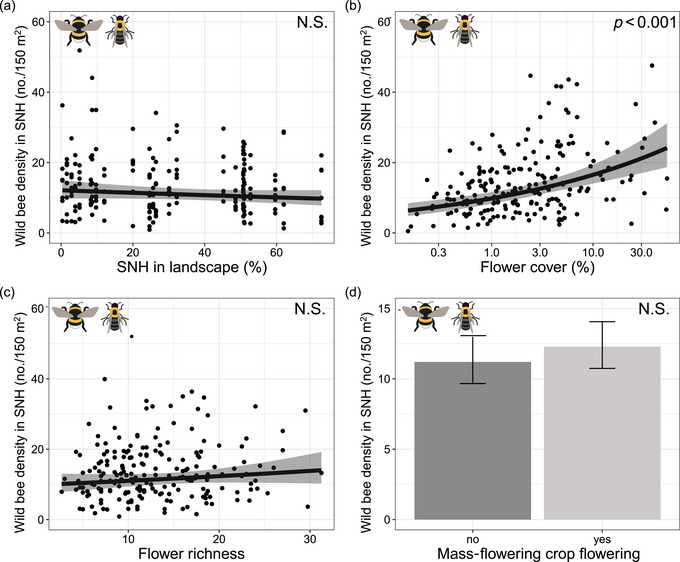
Average wild bee densities in seminatural habitat (SNH) transects in relation to (a) seminatural habitat cover in the landscape in 750‐m radius around landscape center, (b) flower cover (back transformed) in the transect, (c) flower richness in the transect, and (d) whether or not a mass‐flowering crop was flowering in the landscape (N.S., not significant; *y*‐axes, back‐transformed pollinator densities; points, partial residuals; gray shading, 95% confidence intervals).

Wild bee densities in seminatural habitats were not related to the seminatural habitat cover in the landscape (*χ* ^2^_1_ = 1.75, *p* = 0.19) (Figure 1a; Appendix S1), increased with increasing flower cover (*χ* ^2^_1_ = 24.43, *p* < 0.001) (Figure 1b), and were not related to flower richness (*χ* ^2^_1_ = 1.63, *p* = 0.20) (Figure 1c). Whether or not a mass‐flowering crop was flowering did not affect wild bee densities in the seminatural habitats (*χ* ^2^_1_ = 0.72, *p* = 0.40) (Figure 1d), and the effect of the presence of mass‐flowering crops did not depend on seminatural habitat cover (*χ* ^2^_1_ = 0.13, *p* = 0.72).

**FIGURE 2:**
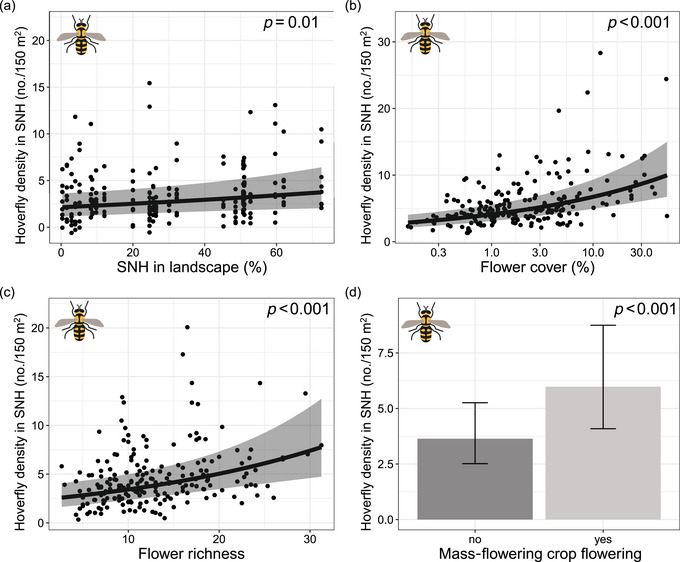
Average hoverfly densities in seminatural habitat (SNH) transects in relation to (a) seminatural habitat cover in the landscape in 750‐m radius around landscape center, (b) flower cover (back transformed) in the transect, (c) flower richness in the transect, and (d) whether or not a mass‐flowering crop was flowering in the landscape (N.S., not significant; *y*‐axes, back‐transformed pollinator densities; points, partial residuals; gray shading, 95% confidence intervals).

Hoverfly densities in seminatural habitats increased slightly as seminatural habitat cover increased in the landscape (*χ* ^2^_1_ = 6.11, *p* = 0.01) (Figure 2a; Appendix S1) and increased strongly as flower cover (*χ* ^2^_1_ = 23.33, *p* < 0.001; Figure 2b) and flower richness (*χ* ^2^_1_ = 14.69, *p* < 0.001) (Figure 2c) increased. When a mass‐flowering crop was flowering, hoverfly densities in seminatural habitats were on average 69% higher than when the crop was not flowering (*χ* ^2^_1_ = 19.03, *p* < 0.001) (Figure 2d) and depended marginally and nonsignificantly on seminatural habitat cover (*χ* ^2^_1_ = 3.61, *p* = 0.06) (Appendix S2).

**FIGURE 3:**
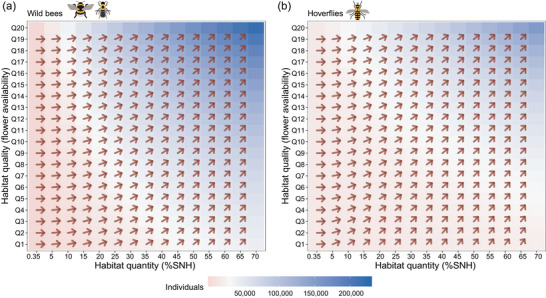
Estimated (a) wild bee and (b) hoverfly population sizes at the landscape level (circular area with a radius of 750 m) as a function of habitat quantity (i.e., percent seminatural habitat cover [SNH]) and habitat quality (flower availability) along the observed ranges in the study (Q1–Q20, 5%‐quantile steps in flower availability, where Q1 is the lowest quality and Q20 is the highest quality; arrows, relative direction of improvements; 0 degrees, increasing habitat quality most beneficial; 90 degrees, increasing habitat quantity most beneficial).

**FIGURE 4:**
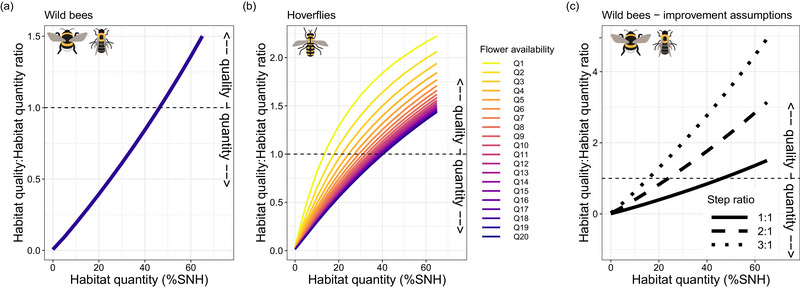
The relationship between the habitat quantity to habitat quality population response ratio, the prevalent cover of seminatural habitat (SNH) in the landscape, and the present habitat quality for (a) wild bees (overlapping lines, present flower cover and richness have little to no influence on the ratio) and (b) hoverflies and (c) the same relationships with different step ratios, with one‐step (solid line), 2‐step (dashed line), or 3‐step (dotted line) increase in habitat quality equaling one step of habitat quantity increase (ratios <1, increasing habitat quantity results in stronger population increases; ratios >1, increasing habitat quality is more beneficial). The same step ratios were applied to hoverflies (Appendix S3).

Extrapolating the observed relationships between pollinator densities and our landscape quantity and quality variables to landscape‐level pollinator population sizes indicated that when there was little pollinator habitat present, improving habitat quantity resulted in the largest increases (Figure 3). As the amount of habitat in the landscape increased, enhancing habitat quality became ever more effective, and this happened relatively sooner for hoverflies than for bees. Assuming that increasing habitat quality is just as feasible as increasing habitat quantity (i.e., 1‐to‐1 step ratio), the threshold at which wild bees benefited more from improvements in habitat quality than in quantity lies at 47% of seminatural habitat cover in the landscape (Figure 4a). For hoverflies, this threshold depended on the prevailing habitat quality. Low‐quality landscapes imposed the threshold at 15% seminatural habitat cover, and high‐quality landscapes imposed the threshold at about 42% seminatural habitat cover (Figure 4b). When assuming other step ratios for wild bees, 2‐to‐1 and 3‐to‐1 ratios decreased this threshold to 24% and 18% seminatural habitat cover, respectively (Figure 4c). For hoverflies, it ranged from 4% to 18% seminatural habitat cover, depending on the prevailing flower availability (lower threshold with lower flower availability) (Appendix S3).

## DISCUSSION

With this study, we have assessed the relative importance of habitat quantity and quality for densities of 2 important pollinator groups in seminatural habitats within agricultural landscapes. We then used these relationships to estimate pollinator population sizes at the landscape scale at different levels of habitat quantity and quality. We found that wild bee densities in seminatural habitats were largely explained by habitat quality (percent flower cover) and not by habitat quantity (percent seminatural habitat cover). Hoverfly densities increased with all our indicators of habitat quality and quantity. At the landscape level, these relationships translated into increasing habitat quantity resulting in the most pronounced increases in pollinator population size when habitat quantity was low to begin with. However, the importance of enhancing habitat quality for boosting pollinator population size increased with increasing habitat quantity.

As expected, wild bee densities in seminatural habitats increased with flower cover (Segre et al., 2023), one of our proxies for habitat quality, but were independent of the surrounding seminatural habitat cover, our proxy for habitat quantity. Results of most studies in agricultural landscapes show that increasing seminatural habitat cover increases pollinator densities, but that is almost exclusively tested in crops (Dainese et al., 2019). There are only a few studies that explicitly assessed the effect of the amount of pollinator habitat on wild bee densities in seminatural habitats (Bartual et al., 2019; Kleijn & van Langevelde, 2006; Li et al., 2020), including an earlier study in the same study region (Fijen et al., 2019), and these studies also showed no such effect. Our results confirmed that wild bee densities in agricultural landscapes are largely determined by the amount of flower resources per surface area (Segre et al., 2023) and therefore that flower availability determines the carrying capacity.

Hoverfly densities increased with higher cover of seminatural habitats, as well as flower availability, making it difficult to assess what determined their carrying capacity. Surprisingly, they also responded positively to nearby flowering crops. Most crop‐visiting hoverflies are highly mobile species (Jauker et al., 2009; Meyer et al., 2009; Wotton et al., 2019) and are known to respond to high resource availability (Kleijn & van Langevelde, 2006; Meyer et al., 2009). The large amount of flowers offered by mass‐flowering crops could attract and concentrate hoverflies from large distances, probably well beyond a few kilometers (Clem et al., 2022; Meyer et al., 2009). Once attracted to the flowering crop, they seem to spillover to seminatural habitats for additional resources. Such attraction and subsequent spillover effect from crop to seminatural habitat is rarely documented (Blitzer et al., 2012) but could mean a (temporary) positive effect of mass‐flowering crop cultivation for hoverfly populations and the pollination and pest‐control services they provide in agricultural landscapes (Dainese et al., 2019).

The extrapolated landscape‐level population estimates suggested that although increasing habitat quantity always had substantial effects on pollinator populations, the importance of improving habitat quality was initially small but became increasingly important as seminatural habitat cover increased. This is logical because of the interplay between habitat quantity and quality at the landscape level. The effect of increases in pollinator habitat on landscape‐level pollinator population size is independent of the amount of habitat that is already present (e.g., a 5% increase in habitat quantity results in a 5% population increase in both simple and complex landscapes). However, increasing habitat quality increased population sizes proportionally to the amount of existing habitat. For example, a 5% increase in habitat quality results in a much larger population increase in complex landscapes with a lot of habitat than in simple landscapes with little habitat (Appendix S4). For hoverflies, this interplay was even stronger than for wild bees because hoverfly densities increased with both flower availability and seminatural habitat cover. Because most agricultural landscapes contain relatively little seminatural habitat, our results generally support the claims for having a minimum amount of seminatural habitat to protect wild pollinator populations (Garibaldi et al., 2020; Kremen & Merenlender, 2018).

To boost pollinator populations, our results suggest that improving habitat quality can represent an alternative strategy for increasing habitat quantity, given that at least 15% of seminatural habitat is present in the landscape (Figure 4; Appendix S3). For example, assuming an agricultural landscape with median habitat quality, a habitat increase from 5% to 10% that is needed to meet the EU Biodiversity Strategy target of 10% of high‐diversity landscape features in 2030 can be matched by roughly 40% (+19.4% flower cover and +12.0 flowering species) and 20% (+4.4% flower cover and +6.0 flowering species) increases in habitat quality for wild bees and hoverflies, respectively. These flower availability improvements are challenging, but they need to be achieved on only 5% of the landscape (i.e., 8.8 ha in our landscapes). However, increasing the seminatural habitat from 15% to the level of 20% that is proposed under the working landscapes principle (Garibaldi et al., 2020; Kremen & Merenlender, 2018) increases the estimated population at the landscape scale roughly as much as a 15% (+2.7% flower cover and +4.5 flowering species) and 10% (+1.5% flower cover and +3.0 flowering species) increase in habitat quality for wild bees and hoverflies, respectively (Appendices S5 & S6). In these more complex landscape contexts, these increases in flower availability seem to be realistic targets that could be achieved through improved vegetation management at the landscape scale, although it can require considerable effort from landowners (Manning et al., 2015; Phillips et al., 2020; Tälle et al., 2016).

We have used a novel and simple method to extrapolate empirical pollinator density data to landscape‐level estimates, which surprisingly has not been done before, likely because of range of logistical issues. Ideally, landscape‐level population sizes are estimated based on transects in as many different (seminatural) habitats and locations within the landscape as possible (e.g., 10–20 transects). One transect takes about 1–2 h, depending on the pollinator and flower communities, limiting the number of transects that can be done on a single day per landscape. Furthermore, to be able to cover a gradient of seminatural habitat cover and flower availability, many different landscapes are required (>15 landscapes). Because the variation in pollinator communities is as large between transect locations as within transect locations over time (Martínez‐Núñez et al., 2022), these transect counts need to be repeated several times within the season. These logistical issues have probably resulted in the lack of studies suitable for estimating pollinator population levels. As far as we are aware, our data set is one of the largest in terms of effort within landscapes (mean 2.8 transects [SD 0.8]), across landscapes (26 landscapes) (but see Alison et al. [2021]), and over time (8 rounds in the full pollinator activity period of 4 months). Because of our large data set, we are confident that our extrapolation to pollinator population at the landscape scale is meaningful, and we show that this method can be a powerful tool to evaluate policy scenarios.

To better conserve pollinator populations in agricultural landscapes, effective conservation measures need to be found. Our study provides support for making decisions on how to increase wild bee and hoverfly populations in agricultural landscapes by showing that in landscapes with <15% seminatural habitat cover, it is most effective to focus on increasing habitat quantity, whereas in more complex landscapes, the focus should be on habitat quality. Ultimately, the choice is largely an economic trade‐off between taking land out of production or managing for improved habitat quality, and future research should focus on the cost‐effectiveness of both pathways and how this trade‐off changes for seminatural habitat types (e.g., hedgerows or field margins [Morandin et al., 2016]). We found that when it is too costly for landowners to take land out of production for creating pollinator habitat, focusing on improving the habitat quality of the non‐productive area under their management can achieve the same results but only when there is substantial amount of seminatural habitat already. Such an alternative is especially important because agricultural landscapes in large parts of the world are under pressure of simplification (Garibaldi et al., 2020), and increasing food security requires that productive land stays in production (Mottaleb et al., 2022).

## AUTHOR CONTRIBUTIONS

**Thijs P.M. Fijen**: Conceptualization; data curation; formal analysis; investigation; methodology; supervision; validation; visualization; writing—original draft; writing—review and editing. **Gabriella A. Bishop**: Conceptualization; validation; writing—review and editing. **Cristina Ganuza**: Data curation; investigation; project administration; writing—review and editing. **Jeroen Scheper**: Conceptualization; methodology; writing—review and editing. **David Kleijn**: Conceptualization; funding acquisition; methodology; resources; supervision; visualization; writing—original draft; writing—review and editing.

## Supporting information

Additional supporting information may be found online in the Supporting Information section at the end of this article.

## Data Availability

The data and code that support the findings of this study are openly available in Zenodo at https://doi.org/10.5281/zenodo.10257700.
