# Supplementary material for: Analyzing the relative importance of habitat quantity and quality for boosting pollinator populations in agricultural landscapes
Source: Conserv Biol. 2024 Jun 25;39(1):e14317. doi: 10.1111/cobi.14317 (PMC11780200; doi:10.1111/cobi.14317)
Supplement: Supplementary file 1 — Additional supporting information may be found online in the Supporting Information section at the end of this article. [file COBI-39-e14317-s001.docx]

**Supporting information “*Analyzing the relative importance of habitat quantity and quality for boosting pollinator populations in agricultural landscapes”***

Appendix S1. Model coefficients from final models for wild bees and hoverflies. P-values are tested using log-likelihood ratio tests (test statistics in main text). All tested two-way interactions were non-significant (p>0.13) and therefore not included here.

| Wild bees | | Parameter estimate | Standard error | z value | p-value (LRT) |
| --- | --- | --- | --- | --- | --- |
|  | Intercept | 0.9685 | 0.0743 | 13.040 |  |
|  | Semi-natural habitat (%) | -0.0013 | 0.0001 | -1.324 | 0.187 |
|  | Flower cover (%; log10-transformed) | 0.2266 | 0.0400 | 5.658 | <0.001 |
|  | Flower richness (# flowering species) | 0.0049 | 0.0039 | 1.277 | 0.202 |
|  | Mass-flowering crop flowering (binary; yes/no) | 0.0403 | 0.0473 | 0.853 | 0.395 |
|  |  |  |  |  |  |
| Hoverflies | | Parameter estimate | Standard error | z value | p-value (LRT) |
|  | Intercept | 0.2875 | 0.1392 | 2.065 |  |
|  | Semi-natural habitat (%) | 0.0025 | 0.0012 | 2.147 | 0.032 |
|  | Flower cover (%; log10-transformed) | 0.2661 | 0.0511 | 5.210 | <0.001 |
|  | Flower richness (# flowering species) | 0.0167 | 0.0047 | 3.533 | <0.001 |
|  | Mass-flowering crop flowering (binary; yes/no) | 0.2159 | 0.0529 | 4.079 | <0.001 |


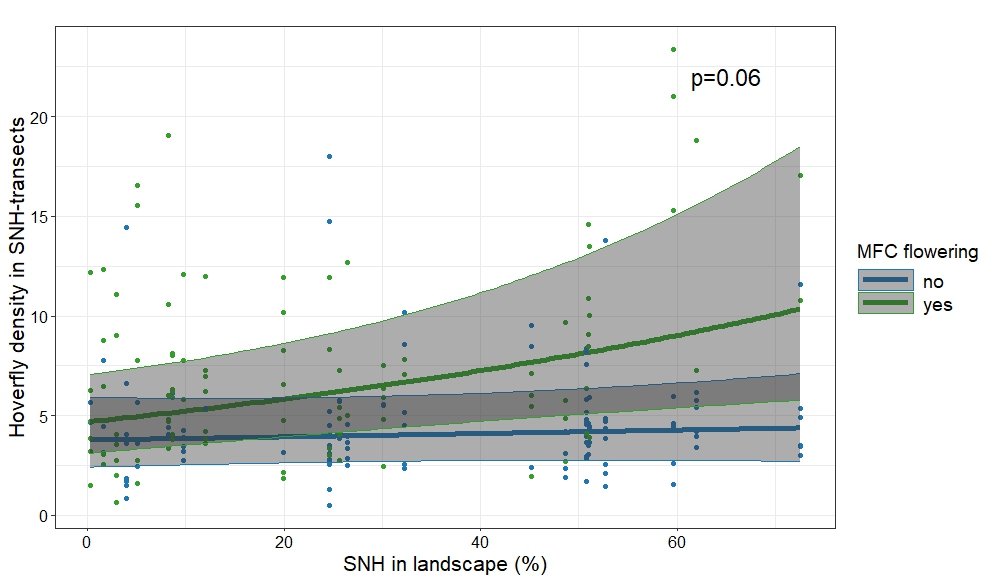


Appendix S2. Average hoverfly densities in seminatural habitat (SNH) transects in relation to seminatural habitat cover in the landscape in 750-m radius around landscape center, depending on whether or not a mass-flowering crop is flowering (blue = no mass-flowering crop flowering, green = mass-flowering crop flowering; interaction is marginally non-significant; y-axes, back-transformed pollinator densities; points, partial residuals; gray shading, 95% confidence intervals).


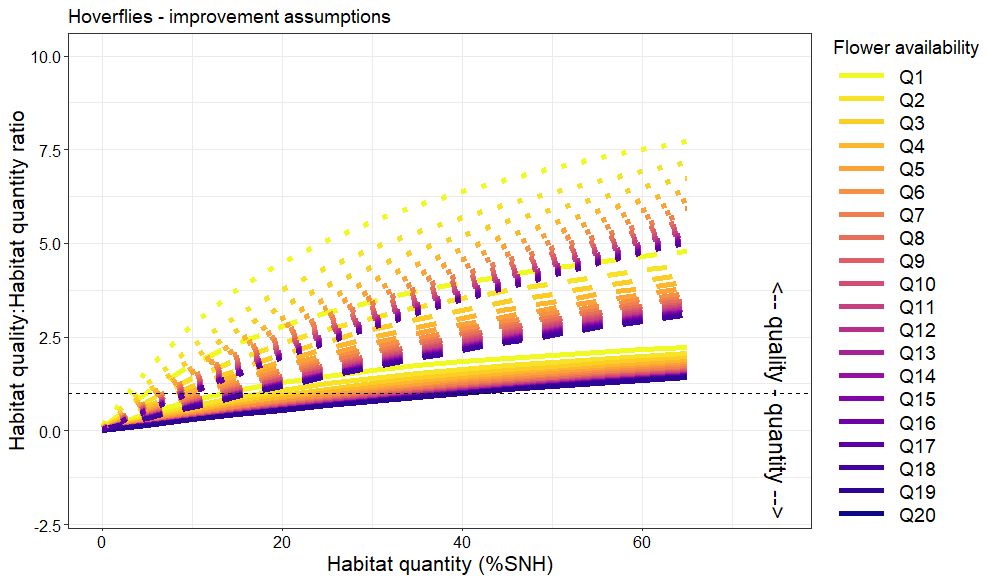


Appendix S3. The relationship between the habitat quantity to habitat quality population response ratio, the prevalent cover of seminatural habitat (SNH) in the landscape, and the present habitat quality for hoverflies. Like in Figure 4c, we varied the step ratio, where one step (solid line), two steps (dashed line) or three steps (dotted line) increases in habitat quality equals one step of habitat quantity increase (ratios <1, increasing habitat quantity results in stronger population increases; ratios >1, increasing habitat quality is more beneficial).


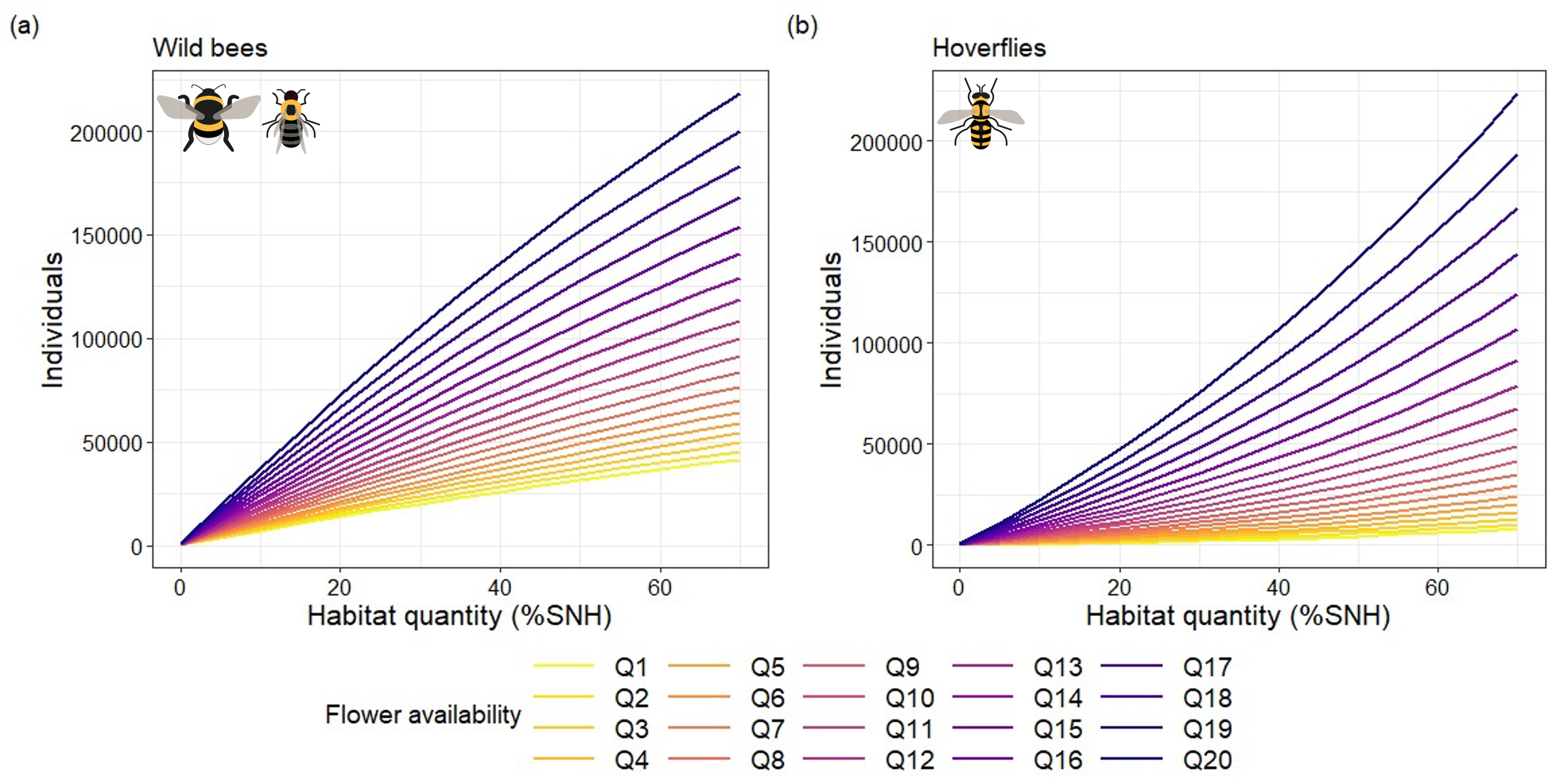


Appendix S4. Estimated population sizes at the landscape level (circular area with a radius of 750 m) for (a) wild bees, and (b) hoverflies (line colour illustrates the habitat quality from yellow, lowest observed, to dark blue, highest observed).

**
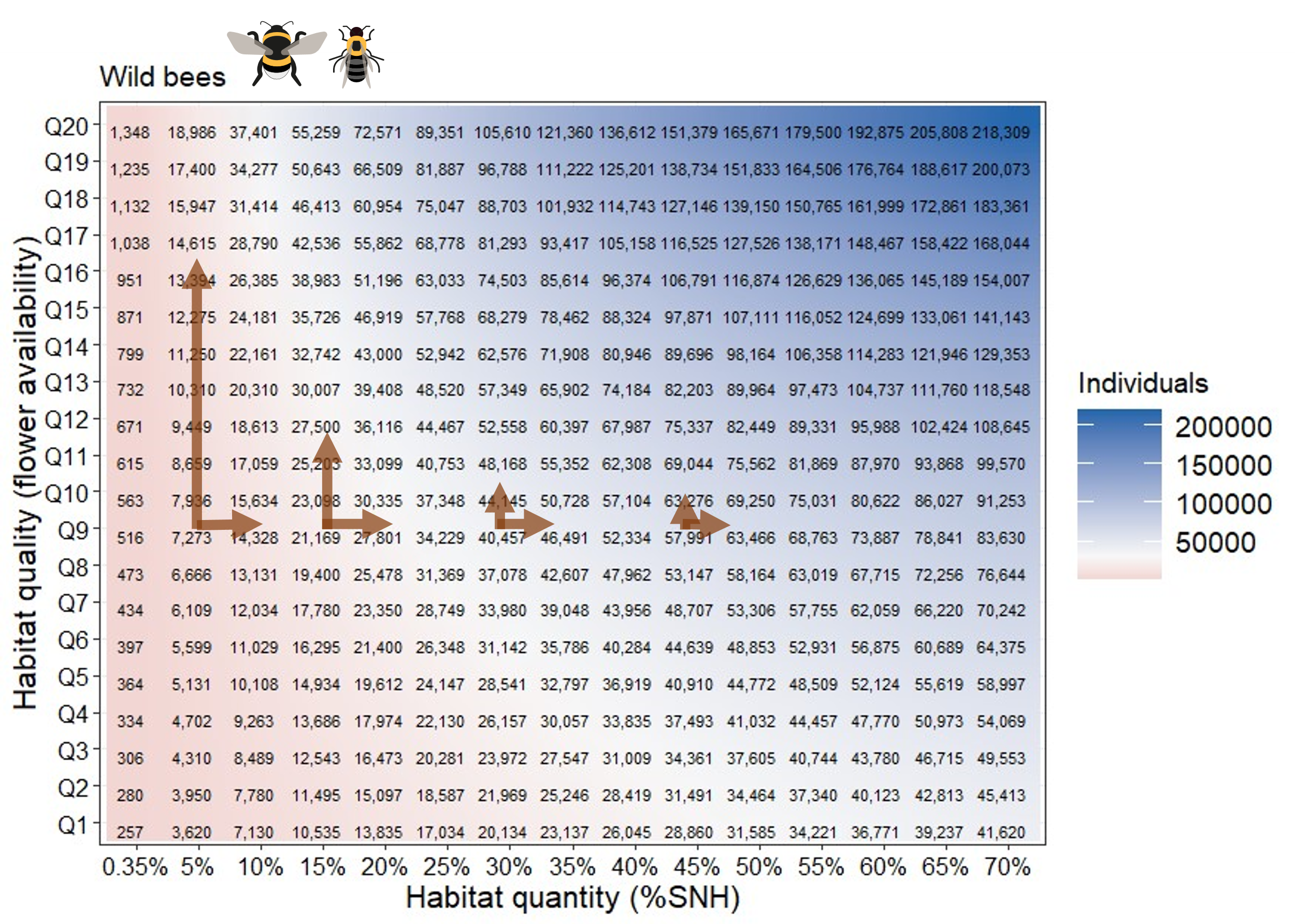
**

Appendix S5. Estimated wild bee population size at the landscape level (circular area with a radius of 750m) as a function of habitat quantity (semi-natural habitat cover (%)) and habitat quality (flower availability) along the ranges actually observed in the study. Q1-Q20 represent 5% steps in flower availability. The arrows illustrate scenarios where, given a median habitat quality (Q9), the vertical arrows indicate how much habitat quality should be improved to have a similar population size of wild bees to the same landscape where 5% more semi-natural habitat is created. The scenario from 5% to 10% illustrates the EU Biodiversity Strategy for 2030 (European Commission & Directorate-General for Environment 2021), the arrow from 15% to 20% a scenario according to the Working Landscape principle (Kremen & Merenlender 2018; Garibaldi *et al.* 2020).


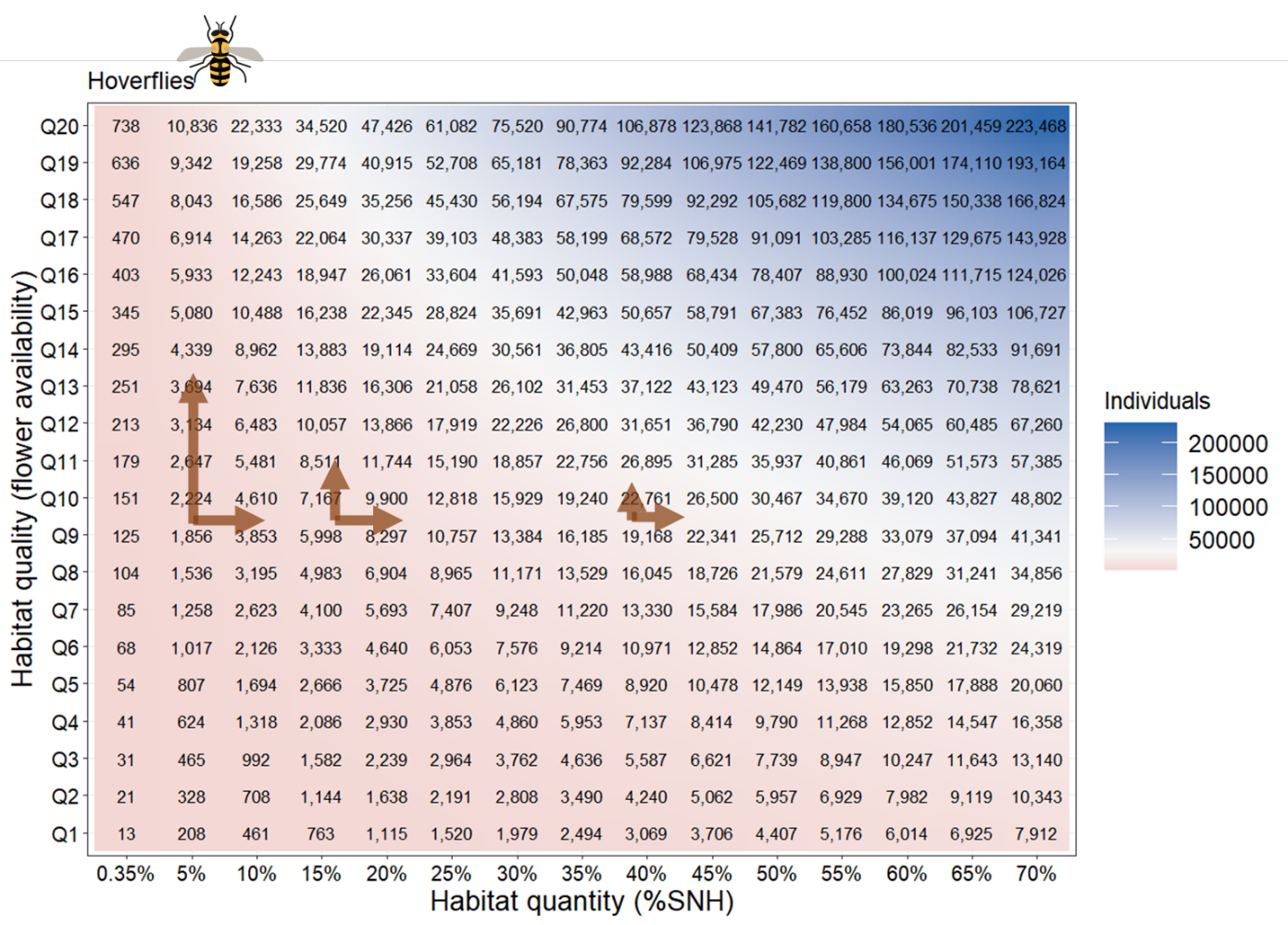


Appendix S6. Estimated hoverfly population size at the landscape level (circular area with a radius of 750m) as a function of habitat quantity (semi-natural habitat cover (%)) and habitat quality (flower availability) along the ranges actually observed in the study. Q1-Q20 represent 5% steps in flower availability. The arrows illustrate scenarios where, given a median habitat quality (Q10), the vertical arrows indicate how much habitat quality should be improved to have a similar population size of hoverflies to the same landscape where 5% more semi-natural habitat is created. The scenario from 5% to 10% illustrates the EU Biodiversity Strategy for 2030 (European Commission & Directorate-General for Environment 2021), the arrow from 15% to 20% a scenario according to the Working Landscape principle (Kremen & Merenlender 2018; Garibaldi *et al.* 2020).

**Supporting table**

**References**

1.

European Commission & Directorate-General for Environment (2021). *EU biodiversity strategy for 2030 : bringing nature back into our lives*. Publications Office of the European Union.

2.

Garibaldi, L.A., Oddi, F.J., Miguez, F.E., Bartomeus, I., Orr, M.C., Jobbágy, E.G. *et al.* (2020). Working landscapes need at least 20% native habitat. *Conservation Letters*, 14, e12773.

3.

Kremen, C. & Merenlender, A.M. (2018). Landscapes that work for biodiversity and people. *Science*, 362, eaau6020.
